# Managing drug shortages in pediatric care

**DOI:** 10.3389/fphar.2024.1416029

**Published:** 2024-06-25

**Authors:** Alexandra Rosário, Balázs Hankó, Romána Zelkó

**Affiliations:** ^1^ Faculty of Pharmacy, University of Lisbon, Lisbon, Portugal; ^2^ University Pharmacy - Institute of Pharmacy Administration, Semmelweis University, Budapest, Hungary

**Keywords:** drug, shortage, pediatric, impact, strategies, systematic review

## Abstract

The global impact of drug shortages on healthcare systems is a concerning issue that needs urgent attention. These shortages not only jeopardize patient care, public health, and healthcare delivery but also pose distinct challenges for pediatric populations due to their specific medication requirements and vulnerabilities. It is imperative to address this issue to safeguard the health and wellbeing of this specific age group. This review Gaimed to conduct a systematic analysis of strategies for addressing drug shortages in pediatric care from 2014 to 2024. The search included five databases: PubMed, Reaxys, Embase, Scopus, and Science Direct, using the keywords “drug shortage” and “pediatric”. The final protocol was developed following the guidelines outlined in the " The PRISMA 2020 statement: An updated guideline for reporting systematic reviews”. In total, 234 publications were identified. After screening the search results and applying inclusion and exclusion measures, a total of 27 original research papers were included. The primary finding indicates that a comprehensive approach rooted in risk management can significantly mitigate drug shortages in pediatric settings. This approach should address underlying causes such as manufacturer and delivery challenges and focus on prevention through enhanced forecasting and vigilant shortage monitoring. The most prevalent response involved seeking alternative treatment options. It is imperative to implement institutional and national guidelines, foster communication, and provider education, and minimize waste to effectively mitigate drug shortages in pediatric settings.

## 1 Introduction

Drug shortages have become a significant concern for healthcare systems worldwide, affecting the delivery of patient care and public health outcomes. While shortages have affected the patient population as a whole, their impact on pediatric populations has been particularly pronounced. Children’s hospitals, in particular, are disproportionately affected, leading to delayed or canceled procedures, changes in care protocols, and the use of less effective alternatives (Addressing pediatric drug and supply shortages, 2024; Pediatric drug shortage trends and best practices for mitigation strategies, 2024).

The scarcity of drugs that affect pediatric populations has become increasingly severe in recent years. This has impacted the treatment of many pediatric disorders. This problem stems from many factors, including production challenges, complex regulations, fluctuating market conditions, supply chain disruptions, financing obstacles, and systemic issues within the healthcare industry (Butterfield et al., 2015; Donnelly et al., 2018; Kelly et al., 2019; Donnelly et al., 2021; Vassal et al., 2021; Addressing pediatric drug and supply shortages, 2024; Pediatric drug shortage trends and best practices for mitigation strategies, 2024).

After examining the impact of medication shortages, it is clear that the lack of essential drugs for children not only delays and reduces the quality of healthcare services, but also creates difficult ethical and practical issues for healthcare providers and organizations. Higher mortality rates, less effective treatments, and the risk of medication errors could result from these issues (Davis et al., 2013; Hughes et al., 2015; Lau et al., 2016; Beck et al., 2017; Lee et al., 2021; Liebman et al., 2023; Rocha et al., 2023).

Given the direct impact of drug shortages on the health and welfare of young patients, it is imperative to study management methods concerning shortages of medication for children. A comprehensive analysis of the current literature is necessary to assess the effectiveness of interventions aimed at mitigating their impact.

Therefore, this systematic review aims to provide a comprehensive overview of the different methods for managing pediatric drug shortages. By conducting a systematic analysis of various literature sources, the review seeks to address the following fundamental question: What can be done to effectively address drug shortages in pediatric medicine, and how can we improve in the future? Through shedding light on the severity of the problem and outlining potential solutions, this review aims to contribute to the improvement of pediatric healthcare and the quality of life of young patients.

## 2 Materials and methods

A systematic literature review has been completed to consolidate findings regarding drug scarcity in the pediatric area. The systematic review protocol was formulated following the systematic review and meta-analysis of PRISMA 2020 guidelines. The search took place in January 2024, with the final run occurring on the twenty-eighth of January 2024.

### 2.1 Data source

The data sources for this systematic review included five electronic databases: PubMed, Scopus, Reaxys, ScienceDirect, and Embase. These databases were chosen to ensure comprehensive coverage of the existing literature on drug shortages in the pediatric field.

### 2.2 Search strategy details

A comprehensive search strategy was meticulously devised with the search terms “drug shortage” and “pediatric”, combined with the Boolean operator “AND” in all databases. A filter has been applied for the time period, which included the years between 2014-2024.

In PubMed, no additional filters were applied. In Scopus, the search was limited to documents classified as “Article” under Document Type. In Reaxys, only articles classified as “Article” under Document Type were included. In Embase, the search was restricted to articles under Publication Type. Lastly, in Science Direct, only research articles categorized as ‘Research Articles’ under Article Type were considered eligible for inclusion."

### 2.3 Eligibility and exclusion criteria

The first inclusion criteria were that the papers must be original peer-reviewed research articles written in English. Only original peer-reviewed research articles were included, while review articles, conference papers, editorials, reports, and commentaries were excluded. Furthermore, articles addressing drug shortages exclusively within the pediatric field were considered. Merely mentioning a drug shortage was insufficient; eligible articles were required to provide detailed data on the shortage, including a comprehensive list of medications in deficit, the duration and location of the shortage, and proposed solutions. It was not enough to mention a general shortage.

### 2.4 Screening process and study selection

A PRISMA 2020 flow diagram was used to extract crucial data for result synthesis. Initially, all database results were exported to Mendeley Reference Manager, and duplicate studies were eliminated.

The screening process involved two stages: initial screening of titles and abstracts and subsequent screening of full-text articles. Screening was conducted by a single reviewer to determine eligibility for inclusion in the systematic review.

During the initial screening stage, titles and abstracts of identified articles were assessed against predefined inclusion and exclusion criteria. Articles deemed potentially relevant were retained for full-text review.

In the second stage, full-text articles selected from the initial screening were examined in detail to determine final eligibility for inclusion in the systematic review. Each article was assessed against the inclusion and exclusion criteria to ensure alignment with the research objectives, with exclusion reasons documented. There were categories created on Mendeley Reference Manager to help sort the excluded articles by reason. Supplementary Figure S1 displays the PRISMA flowchart illustrating the screening results, paper selection process, and rationale for exclusions.

## 3 Results and discussion

The literature search identified two hundred and thirty-four (*n* = 234) in the first round of analysis, among which twenty-eight (*n* = 28) from PubMed, fifty-five (*n* = 55) from Scopus, forty-five (*n* = 45) from Reaxys, seventy-nine (*n* = 79) from Embase, and twenty-six (*n* = 26) from Science Direct.

One hundred and twenty-nine (*n* = 129) articles remained after removing duplicate articles. Forty-eight (*n* = 48) duplicates were removed using Mendeley Reference Manager and seventy-two (*n* = 72) by human hand.

After reading the titles and abstracts, another sixty-one (*n* = sixty-one) articles had to be excluded because they did not meet the inclusion criteria. Out of these total, forty-one (*n* = 41) were excluded after screening the full texts.

In the end, twenty-seven (*n* = 27) studies met all the criteria selected for the review. A flowchart of the search is illustrated in (Page et al., 2021).

### 3.1 Study characteristics

The systematic review included 27 studies conducted across various countries globally. These studies spanned America (USA (United States), Brazil, Canada, and the Caribbean - The Bahamas, Barbados, Jamaica, and Trinidad and Tobago), several countries in Europe (Belgium, Bulgaria, Croatia, Slovenia, Finland, Greece, Italy, Spain, Sweden, Switzerland, United Kingdom, Norway, Denmark, Irelan, Sweden, Netherlands, Austria, Germany, France, Israel, Cyprus, Malta, Portugal, Estonia, Czech Republic, Lithuania, Latvia, Hungary, Poland, Romania, Turkey, and Serbia), the East Mediterranean, the Western Pacific, Asia (Taiwan and Thailand), and Africa (Kenya and Ghana).

Most of the research papers were data analysis based on retrospective studies and surveys. Additionally, almost all the research was carried out in pediatric health facilities, for both inpatient admission and outpatient clinics, between 2001 and 2023. All the studies mentioned the shortage product(s), while some concentrate on one or few substances in scarcity, others address dozens.

Supplementary Table S1 summarizes these characteristics according to each study, by shortage drug(s).

### 3.2 Qualitative synthesis outcomes

The research papers included in this review address effective strategies for managing drug shortages, that have been successfully utilized in diverse pediatric healthcare settings or proposed based on the research ensured. Supplementary Table S2 demonstrates the proposed strategies by study and drugs in shortage.

### 3.3 Strategies for managing drug shortages

Managing drug shortages in pediatric care requires a comprehensive approach that involves various strategies to ensure the best possible care for patients. Supplementary Table S3 highlights the key points and outcomes of different strategies used to address drug shortages in pediatric care. Each strategy has its unique key points and outcomes, which are summarized in the table below.

#### 3.3.1 Drug supply and distribution

Pediatric care facilities often face medication shortages due to manufacturing, transportation, and supply and demand issues. As a result, mitigation strategies are employed, such as seeking alternative sources and giving financial incentives to suppliers to improve drug distribution processes (Vassal et al., 2021; Donnelly et al., 2018; Donnelly et al., 2021; Salazar et al., 2015; Kontturi et al., 2016; Ziesenitz et al., 2018; Kanja et al., 2021; Chen, 2017; Claus et al., 2018; Boateng et al., 2021; Bauters et al., 2015; Lee et al., 2023; Kremer et al., 2023).

In a study by Goodman et al. (2015), obtaining the missing drugs from other institutions was the prevalent institutional strategy used for combatting chemotherapy shortages, being reported by 66%. The significant proportion of pharmacist respondents who reported obtaining a drug in shortage from another institution implies that such requests are sometimes successful, despite the fact that institutions are generally reluctant to release drugs in shortage due to the need to treat patients within their own facilities.

In the same paper, purchasing alternative vial sizes was another alternative solution proposed to a drug shortage that pharmacists may use. Introducing such action can lead to added complexities such as the need for safety training, physical stocking adjustments, and updates to electronic medical records. It is essential to make adaptations to maintain medication supply, but these changes can cause operational challenges. Therefore, careful management of drug stock, operational and personal adjustments, and thorough attention to additional expenses are crucial to ensure both patient safety and continuous care provision in this cases (Salazar et al., 2015).

In the research by Kontturi et al., 2016, authorities in two countries considered changing the manufacturer/supplier to address the shortage of the Bacille Calmette-Guérin vaccine (Kontturi et al., 2016).

Donnelly et al., 2021 proposed that manufacturers should improve their quality control processes to avoid delays and shortages caused by manufacturing and quality issues. They also recommended diversifying manufacturing sources to decrease the risk of shortages due to problems at a single facility. Furthermore, offering incentives for manufacturers to produce drugs in short supply can help address shortages (Donnelly et al., 2021).

According to Donnelly et al., 2018, the American Academy of Pediatrics suggested implementing additional measures to protect children from medication shortages. These measures included ensuring fair distribution of drugs during shortages, conducting more research on pediatric drugs, and improving their proper labeling (Donnelly et al., 2018).

In a study by Ziesenitz et al., 2018, it was suggested that importing drugs should be considered as a measure to tackle drug shortages on a national level. However, it is important to note that importing medications from other countries should be done only when the source country has a surplus to avoid creating shortages elsewhere. Nevertheless, it is crucial to exercise caution to ensure the safety and quality of substitutes, especially when it comes to vulnerable populations such as neonates. On a provider network level, expanding current suppliers and implementing flexible supply strategies were recommended (Ziesenitz et al., 2018).

Causes of stockouts of vaccines include unavailability of products, unreliable deliveries, and lack of transport. Kanja et al. (2021) highlighted the significance of proper planning for vaccine delivery. To prevent vaccine shortages and ensure timely vaccination, it is crucial to ensure proper storage and transportation, enhance logistics, and strengthen delivery systems. They emphasized the importance of having reliable delivery systems and training the supply chain officers to maintain vaccine potency (Kanja et al., 2021).

In a study by Chen, 2017, it was demonstrated that sharing and redistributing influenza vaccines can increase their availability and reduce unused vaccines during seasonal influenza interventions. Sharing enables different age groups to exchange vaccine types, while redistribution involves moving vaccines between geographic regions to match supply with demand. Sharing pediatric vaccines can reduce vaccine unavailability by 43% and overstock by 54%, and sharing adult vaccines can reduce unavailability by 9% and overstock by 15%. Redistributing vaccines can result in even greater gains (up to 75%) (Chen, 2017).

Claus et al., 2018 discussed solutions for drug shortages, including purchasing generics, searching for another national (Belgium) provider, and importing the drug from other European Union countries. In case of critical shortage, it suggested purchasing from alternative producers or hospitals. However, drug shortages can increase prices, and importing from international wholesalers may not be covered by national health insurance, affecting patient invoices (Claus et al., 2018).

According to Boateng et al., 2021, enhancing supply chain efficiency is crucial. This involves tackling inefficiencies within the supply chain that hinder the consistent and sufficient availability of medications for childhood cancer. It also highlighted that country-level supply chain inefficiencies play a major role in limiting the availability and affordability of these drugs (Boateng et al., 2021).

Bauters et al., 2015 found that drug shortages can be addressed by purchasing generic alternatives or importing drugs from other countries, both of which involve seeking alternative suppliers. They observed that in over one-third of cases, a generic alternative was purchased (35.2%) and in 14.8% of cases, drugs were imported from abroad. Obtaining drugs from another institution is also mentioned as a common solution to handle drug shortages (Bauters et al., 2015).

The research by Lee et al., 2023 emphasizes the importance of improving supply chain systems for enhancing infant vaccination coverage in low- and middle-income countries. The authors have also discussed global efforts, such as the Global Vaccine Action Plan and Immunisation Agenda 2030, which aim to protect children from preventable diseases by improving supply chain systems and ensuring universal access to vaccines (Lee et al., 2023).

A less common method to manage shortages was addressed in a paper by Kremer et al. (2023). The study found that using drones for vaccine delivery led to significant improvements in the vaccine supply chain, program performance, and clinical outcomes in the Western-North Region of Ghana. The researchers concluded that end-to-end aerial logistics is an effective tool for enhancing the vaccine supply chain, increasing vaccination coverage, and improving satisfaction among healthcare providers in the region (Kremer et al., 2023).

The paper by Vassal et al., 2021 discusses incentives for improving production infrastructure, including financial incentives to address the economic causes of manufacturing issues and incentives for suppliers to remain in the markets to resolve shortages (Vassal et al., 2021).

#### 3.3.2 Therapeutic alternatives

Changing therapeutic schemes has been revealed to be of great importance regarding the management of drug scarcities. The substitution for replacement drugs (e.g., second-line agents), dosage forms, dosage strengths, or different treatment plans was proposed in several research articles. It was highlighted for having a substantial impact on maintaining an effective therapy when in lack of the standard treatment (Donnelly et al., 2018; Donnelly et al., 2021; Hughes et al., 2015; Vagrecha et al., 2023; Rocha et al., 2021; Barnes et al., 2020; Salazar et al., 2015; Brewster et al., 2023; Ziesenitz et al., 2018; Diachinsky et al., 2021; Visage et al., 2019; Nickel et al., 2013; Claus et al., 2018; Mangum et al., 2020; Patel et al., 2020).

In the article by Vagrecha et al., 2023, *Erwinia* asparaginase was in shortage, which represented a problem for patients who develop a hypersensitivity reaction to the pegylated form of *E. coli* derived asparaginase. As a result, they used a premedication strategy, followed by desensitization in patients who needed it, as an alternative. They only used *Erwinia* asparaginase in patients in which the previously mentioned treatment did not work (Vagrecha et al., 2023).

In a study by Rocha et al., 2021, many of the patients received an altered treatment scheme, with penicillin being replaced with ceftriaxone and cefazolin, in most cases, or a combination of medications (Rocha et al., 2021).

According to Barnes et al., 2020, fentanyl and hydromorphone were the primary intravenous opioids utilized in response to the limited availability of morphine during the 2018 shortages. An increased use of adjuvant pain medications, including acetaminophen and nonsteroidal anti-inflammatory drugs was also seen, but not significantly (Barnes et al., 2020).

In a paper by Goodman et al., 2015, one of the most common institutional methods of addressing drug shortages included changing patient regimens (39%) (Salazar et al., 2015).

Brewster et al., 2023 explored an amoxicillin shortage and its decrease in prescriptions and noted that it coincided with a rise in the utilization of alternative agents such as amoxicillin-clavulanate and cefdinir as treatment options for Acute Otitis Media. The article also mentioned the recommendation by The American Academy of Pediatrics of splitting tablet or capsule formulations to substitute the formulation missing in the treatment (Brewster et al., 2023).

Donnelly et al., 2021 discussed the need for formulation substitution during shortages, which involved using alternative formulations of medications to improve the availability of childhood cancer medicines. This is particularly important in the context of stockouts of specific vial sizes. The research also brought attention to issues regarding inadequate vial sizes for typical pediatric dosages, along with possible delays in providing medication if pharmacists were not present for reconstitution (Donnelly et al., 2021).

The study by Donelly et al., 2018 found that 86% of drugs facing shortages had alternatives. However, 29% of these alternatives were also impacted by shortages. This highlights the need for healthcare providers to stay informed about shortages and develop strategies to optimize patient care (Donnelly et al., 2018).

Ziesenitz et al., 2018 suggested using substitutes or adapted therapies during drug shortages at the hospital level, particularly for less vulnerable patients. However, not all alternative drugs have been tested in the pediatric population, so this strategy may not be applicable in all cases. Alternative administration routes were also recommended, but their use in the neonatal intensive care unit is limited to non-life-saving indications. It is important to conduct a daily assessment of patients’ current treatment and available options (Ziesenitz et al., 2018).

The study by Nickel et al., 2013 used mitoxantrone as a substitute for daunorubicin during a shortage, in leukemia and lymphoma treatments (Nickel et al., 2013).

In Hughes et al., 2015, a shortage of fentanyl and intravenous benzodiazepines was studied and alternative therapies for pain and sedation management were recommended by clinical pharmacists and critical care physicians. These included the use of different opioids like morphine and hydromorphone, as well as less common sedatives such as propofol, pentobarbital, ketamine, and dexmedetomidine (Hughes et al., 2015).

In Claus et al., 2018, the change of ongoing treatments was used by 2.4%, only for parenteral drugs, such as melphalan and polyvalent immunoglobulins (Claus et al., 2018).

In the paper by Diachinsky et al., 2021, oral sodium bicarbonate with lactated Ringer’s was used as a feasible alternative for urine alkalinization during an intravenous sodium bicarbonate shortage in patients going through high-dose methotrexate (Diachinsky et al., 2021). For the same shortage and treatment, in Visage et al., 2019, patients also received oral sodium bicarbonate in the form of tablets or sodium citrate-citric acid oral solution as alternatives to mitigate nephrotoxicity (Visage et al., 2019). Mangum et al., 2020, on the other hand, addressed the sodium bicarbonate drug shortage, by altering the institutional standard treatment supportive care guidelines. During the shortage, patients received alternative IV hydration and urinary alkalinization methods, such as enteral sodium bicarbonate, enteral sodium citrate, bolus parenteral sodium bicarbonate, or a combination of these. This changed the pre-shortage standard of care, using a different IV fluid composition and urinary alkalinization methods compared to the standard practice (Mangum et al., 2020).

In the research developed by Patel et al., 2020, cefotaxime was replaced by ceftazidime during a national shortage in the United States (Patel et al., 2020).

Bauters et al., 2015 change in individual treatment plans were addressed as a solution to drug shortages, for example, the need to switch from vindesine to vincristine treatment (Bauters et al., 2015).

A less used approach, mentioned in a study by Brewster R. et al., 2023, is watchful waiting. This involves delaying therapeutical intervention and closely monitoring a medical condition while waiting for the drug to be available again (Brewster et al., 2023).

When choosing an alternative treatment option during a shortage, several factors are important to consider. Ideally, the decision must be based on efficacy and toxicity, aiming for equal or better effectiveness and less toxicity to improve patient outcomes, while minimizing side effects (Bauters et al., 2015; Brewster et al., 2023). Substitute drugs and alternative formulations may also suffer from short supply due to their overuse to address another shortage. Therefore, this method should be used carefully and monitored closely.

#### 3.3.3 Compounding pharmaceutical formulations

This strategy aims to customize medicines to address the drug shortage needs. It consists of altering available products to create personalized medications that are not readily available on the market at that moment. When a medication is in short supply and it has different dosage forms, it can be possible to change the available formulation to the one lacking. Compounding adult forms into child-friendly forms is also possible (Barnes et al., 2020; Salazar et al., 2015; Ziesenitz et al., 2018; Claus et al., 2018; Tuesuwan et al., 2022).

In fact, in a study by Barnes et al., at a children’s hospital, pharmacists were trained to incorporate opioid intravenous to per os conversion into their workflow and everyday schedule in the pharmacy services (Barnes et al., 2020).

This method is one of the most used ones in case of a shortage. According to Goodman et al., 2015, converting the missing drug to another form was the second most common method to address the shortages at an institutional level (Salazar et al., 2015).

Despite being frequently used, this strategy is not always easy to execute. Ziesenitz et al. (2018) have noted that creating pediatric formulations through compounding of adult preparations can lead to incorrect dosages and increased risks of microbial contamination due to the need for additional dilution. Even so, this is one of the used mitigation strategies for drug shortages in this article, at a hospital and ward level. Preparing compounded drugs in the hospital pharmacy and using them for less vulnerable patients, such as adults, can be very helpful, while the standard drug treatment in shortage is preserved only for the sickest patients (Ziesenitz et al., 2018).

In the paper by Claus et al., 2018, pharmaceutical preparation or modification in the hospital was sometimes required for oral drugs (phenoxymethylpenicillin and ranitidine), particularly for children with specific dosage needs. This approach, although protocol-driven and labor intensive, offered a potential solution (30).

According to Tuesuwan et al., 2022, during the COVID-19 crisis there was a shortage of favipiravir for pediatric patients. To address the problem, favipiravir was formulated from a tablet form to an oral solution for hospital compounding. This oral solution was developed as a child-friendly dosage form to improve dosing accuracy and patient compliance, especially for pediatric patients (Tuesuwan et al., 2022).

#### 3.3.4 Inventory management and waste reduction

During times of drug shortages, and even beforehand, it is crucial to have an effective inventory management system in place. While finding alternatives to drugs in shortage can be helpful and reduce the usage of the drug needed, it is not always sufficient as it may lead to new shortages of the substitutes. Minimizing waste, using medications for all patients who need them at the same time, and avoiding hoarding can make a significant difference in saving and managing the existing stock (Liebman et al., 2023; Hughes et al., 2015; Salazar et al., 2015; Kontturi et al., 2016; Ziesenitz et al., 2018; Hemmann et al., 2022; Boateng et al., 2021).

The paper by Goodman et al., 2015 mentioned the reduction of dosage use during chemotherapy treatment as one of the methods applied at an institutional level to resolve the shortage problems (Salazar et al., 2015).

In the article by Kontturi et al., 2016, cohorting vaccinees is a recommended action to address drug shortages. Grouping vaccines on specific days to use multi-dose vaccine vials has been proven effective in reducing vaccine consumption. For example, in Finland it resulted in a 50% reduction in vaccine consumption. Australia, on the other hand, had a high vaccine wastage rate of 75%. The WHO (World Health Organization) recommends opening multi-dose vials for any vaccination session size and calculating wastage based on doses per vial and daily vaccinations. It also suggests procuring vaccines based on expected demand with a 25% buffer for open vial wastage (Kontturi et al., 2016).

In the same paper, stockpiling vaccines was also an action taken. This strategy involves storing vaccines in advance to effectively respond to shortages, outbreaks, epidemics, and emergencies at local, regional, or global levels (Kontturi et al., 2016).

According to Ziesenitz et al., 2018, during drug shortages, treating patients who require the same medication simultaneously or within the drug’s shelf-life after preparation is a practical strategy that reduces drug waste. Additionally, it is recommended to avoid stockpiling or excessive purchases and to carefully review the available products. A less conventional strategy would be to use expired medications, but only after obtaining regulatory approval (Ziesenitz et al., 2018).

In Donnelly et al., 2021, proposed that stockpiling of critical drugs can help ensure availability during shortages (Donnelly et al., 2021).

In a study conducted by Hemmann et al., in 2022, the researchers investigated the impact of early fortification while reducing throphamine usage during a shortage. This required a modification of feeding guidelines to make the necessary adjustments. The study concluded that this approach did not result in malnutrition, allowing the saved throphamine to be distributed to more critically ill children (Hemmann et al., 2022).

Hughes et al., 2015 created a standardized method to save and manage the supply of medications that are in short supply. The clinical pharmacists developed printed materials that contain step-by-step instructions for usage, recommended dosages, and monitoring parameters for alternative drugs (Hughes et al., 2015).

According to the article by Liebman et al., 2023, during a *haemophilus* influenzae type b vaccine shortage, delaying booster doses in favor of primary series doses was implemented. It can be an effective strategy for managing vaccine distribution and ensuring that the most vulnerable populations receive necessary vaccinations during a vaccine shortage. However, it is crucial to consider the potential consequences of delayed booster doses, such as the need for catch-up vaccination and the possibility of incomplete or inaccurate vaccine records (Liebman et al., 2023).

#### 3.3.5 Patient cohorting and prioritization

In times of drug shortages, healthcare facilities may opt to prioritize certain patients, based on individual requirements and urgency. It may be necessary to evaluate which patients truly require medications in shortage and those who can receive second-line treatments or alternatives. This decision-making process is complex and may require guidelines or medical boards to reach a conclusion.

Cohort patients is a strategy frequently mentioned in the articles reviewed, as it enables efficient management of available medications during shortages. This involves grouping patients based on specific criteria, such as their medical needs or the urgency of their care. This practice helps healthcare providers prioritize patients based on their clinical requirements and optimize the allocation of limited drug supplies to those who need them most urgently (Hughes et al., 2015; Rocha et al., 2021; Salazar et al., 2015; Kontturi et al., 2016; Ziesenitz et al., 2018; Claus et al., 2018; Bauters et al., 2015).

According to Rocha et al., 2021, newborns who show symptoms of congenital syphilis at birth, premature infants, those born with low birth weight, or with specific medical conditions are more likely to receive standard penicillin treatment. On the other hand, those who do not fall into these categories are treated with alternative methods, as mentioned before (Rocha et al., 2021).

Goodman et al., 2015 found that almost half (47%) of healthcare facilities use the practice of scheduling patients to receive a scarce drug on the same day to reduce waste. The reasons for not using this method are unclear. Some facilities find it unnecessary or impossible, while others may not do it due to billing requirements (Salazar et al., 2015).

In a study by Kontturi et al., 2016, strategies were used to minimize Bacille Calmette-Guérin vaccine shortages by targeting high-risk individuals. Spain and the United Kingdom administered the vaccine selectively to high-risk groups due to limited availability (Kontturi et al., 2016).

In Ziesenitz et al., 2018, during drug shortages affecting medications used in the NICU, the use of substitutes or compounded formulations was proposed only for non-priority groups of patients. Conversely, the prioritization of the most critically ill patients, such as extremely low birth weight infants, was recommended to be implemented at the ward level within the hospital. This article recommends using only pediatric/neonatal formulations only on these patients to mitigate shortages at the hospital level (Ziesenitz et al., 2018).

In the research by Hughes et al., 2015, when a shortage of fentanyl and intravenous benzodiazepines happened, certain patient groups were given priority access, and restrictions were placed on the use of those medications in the Pediatric Intensive Care Unit. Specifically, fentanyl and midazolam were preferentially prioritized for cardiovascular surgery and cardiac intensive care unit patients. This is one of several approaches recommended by various organizations for managing drug shortages (Hughes et al., 2015).

According to Claus et al., 2018 high-impact solutions which influenced physician practices and patient clinic visits, like cohorting of patients were targeted for parenteral treatments, notably melphalan and polyvalent immunoglobulins (Claus et al., 2018).

In a study by Bauters et al., 2015, during a chemotherapy drug shortage at a tertiary care hospital, the institution opted to prioritize or cohort a specific medication for specific patient populations. For example, when facing a shortage of amsacrine, the hospital decided to allocate this drug to adults rather than children (Bauters et al., 2015).

When demand exceeds supply, ethical dilemmas surface. International guidelines assist in informed decision-making, yet complexities arise in pediatrics due to off-label drug use. Allocating drugs for both pediatric and adult care poses challenges and focusing solely on the pediatric population can also lead to decision-making complexities (Bauters et al., 2015).

#### 3.3.6 Forecasting and shortage monitoring

Improving the way we predict and monitor drug shortages can help us be better prepared. We can do this by identifying the factors that cause these shortages, such as the type of drug, what it treats, and how many manufacturers produce it. By taking a proactive approach, pharmacies, drug manufacturers, and suppliers can address and mitigate drug shortages before they happen. This helps to reduce the extra work and stress caused by drug shortages and allows for better planning and allocation of medication.

In order to effectively address drug shortages, it is also essential to have real-time and effective monitoring and alerts in place. This allows for greater transparency in the supply chain, enabling quicker identification of potential shortages and immediate action to be taken to prevent or mitigate any impact. By implementing such systems, healthcare providers and patients can have greater confidence in the availability of essential medications, ensuring that they receive the care they need in a timely and effective manner (Donnelly et al., 2018; Ziesenitz et al., 2018; Boateng et al., 2021; Kanja et al., 2021; Vassal et al., 2021).

In Donnelly et al., 2018, providers working in the ambulatory setting must be aware of current shortages, advanced notification allows the FDA (Food and Drug Administration) to ask other suppliers to increase production, expedite reviews, or take other actions to prevent shortages. It may be that the FDA’s efforts regarding early notification about shortages may be improving time to resolution (Donnelly et al., 2018).

Ziesenitz et al. (2018) stated that since 2012, manufacturers have been required to notify the FDA of anticipated product shortages or discontinuations. Consequently, there was a decline in newly occurring drug shortages in 2012. The article emphasizes the importance of early information about anticipated drug shortages on a national level and urges the anticipation of drug shortages at a regional level (Ziesenitz et al., 2018).

According to a study by Kanja et al., 2021, inadequate forecasting is a significant contributor to drug stockouts. To prevent vaccine shortages and ensure timely vaccination, it is critical to enhance vaccine forecasting through collaboration of supranational governance, regulatory bodies, and national institutions to forecast and monitor medicine supply. Comprehensive training programs should be organized on vaccine forecasting at the sub-national level and health facilities to facilitate effective planning, avoid stockouts, and ensure vaccine availability (Kanja et al., 2021).

In a paper by Boateng et al., 2021, it is suggested that evidence-based drug forecasting could aid in better planning and allocation of drug budgets, earmarking inventories for pediatric cancer, and negotiations with international manufacturers to alleviate overall drug shortage problems. This strategy should be implemented by institutions responsible for supply chain logistics, drug management, national and local authorities, and health workers trained in vaccine handling and management (Boateng et al., 2021).

The article by Vassal et al., 2021 emphasizes the importance of enacting legislation to provide early alerts for shortages of essential anticancer medications for children and adolescents. This would ensure timely access to these crucial medications for pediatric patients across Europe. Another recommendation is the creation of national essential medicines lists based on the WHO’s Essential Medicines List, customized to meet the specific needs of each country. Additionally, establishing a database to monitor shortages, with a common set of data requirements for all countries, can help prevent and monitor drug availability (Vassal et al., 2021).

#### 3.3.7 Guidelines and policies

To prevent and mitigate future shortages of essential medications worldwide, it is important to establish effective international policies and guidelines. By doing so, we can ensure that a stable and reliable supply of these medications is maintained, which is crucial for promoting the health and wellbeing of people around the globe. These policies should be carefully developed and implemented, taking into account the unique challenges and needs of different regions and populations, in order to maximize their effectiveness and overall impact (Donnelly et al., 2018; Donnelly et al., 2021; Barnes et al., 2020; Salazar et al., 2015; Ziesenitz et al., 2018).

In the study by Barnes et al., 2020, the Children’s National Medical Center implemented an opioid stewardship program to address opioid shortages in late 2017. To achieve this, a task force was created to integrate opioid intravenous to per os conversion alerts into daily workflow, educate providers, and send weekly shortage emails. The program successfully managed pain without increasing medication errors. It also highlighted the importance of multimodal pain control and appropriate pharmacy education (Barnes et al., 2020).

According to Goodman et al., 2015, pediatric institutions have started implementing recommendations to address drug shortages, such as issuing center-level guidelines. According to a survey, around 30% of adult hospitals have center-level guidelines to address shortages. However, in pediatric institutions, 73% of pharmacist respondents reported having center-level guidelines. This indicates that there is a high percentage of pediatric centers with pharmacy guidelines to address drug shortages, which might be an effective action to take (Salazar et al., 2015).

In the research by Donnelly et al., 2018, The American Academy of Pediatrics measures to protect children from drug shortages are mentioned. They include creating a list of critical pediatric drugs (Donnelly et al., 2018).

The article by Ziesenitz et al., 2018 advises following the recommendations and guidelines of professional societies such as American Academy of Pediatrics, American Society for Parenteral and Enteral Nutrition, American Society of Health-System Pharmacists, and Children’s Oncology Group based on the specific drug (Ziesenitz et al., 2018).

In Donnelly et al.’s 2021 study, they discuss the expansion of National Essential Medicines Lists to include a wider variety of cancer medications specifically for childhood cancer treatment (Donnelly et al., 2021).

The paper by Vassal et al., 2021 mentions recommendations to address shortages of inexpensive essential medicines. It emphasizes the importance of establishing policies and plans to better manage shortages and lack of accessibility to drugs in pediatric settings in Europe. One proposed strategy is to establish legislation for early notifications of shortages. Additionally, it is suggested to develop European strategic plans for medicine shortages and to establish procurement models for acquiring and managing essential medicines. These methods aim to prevent shortages, ensure financial accessibility, and improve the availability of safe and effective medications for pediatric cancer patients (Vassal et al., 2021).

#### 3.3.8 Collaboration, communication and education

During times of drug shortages, national health agencies, manufacturers, healthcare providers, clinical pharmacists, and critical care physician leadership need to work together to ensure that patients receive the best possible care. To do this, healthcare providers should be trained on drug shortages, and the entities involved must communicate constantly to share best practices and find solutions. It is also important for national and county governments to work together to streamline their responsibilities and provide an efficient response to drug shortages. When everyone works together, it is easier to make sure that patients get the medicines they need (Kontturi et al., 2016; Barnes et al., 2020; Donnelly et al., 2021; Kanja et al., 2021; Hemmann et al., 2022; Roth et al., 2024).

In a study conducted by Barnes et al., 2020, the institution being researched implemented provider education as a strategy during an opioid shortage. This approach is believed to have prevented higher rates of uncontrolled pain or increased adverse events, when coupled with appropriate pharmacy education (Barnes et al., 2020).

The survey in Kontturi et al., 2016 emphasizes the importance of collaboration between national health agencies and vaccine manufacturers to ensure necessary Bacille Calmette-Guérin immunizations and address the shortage effectively. The collaboration between national health agencies and vaccine manufacturers is vital for ensuring vaccine availability, quality, and effective distribution. This partnership addresses challenges like shortages, quality assurance, and policy development, aligning immunization programs with global standards. International coordination with organizations like WHO and UNICEF enhances efforts to address shortages and promote equitable vaccine access globally, crucial for maintaining effective immunization practices and global health security (Kontturi et al., 2016).

The article by Roth et al., 2024 discusses the implementation of a tier system for intravenous immune globulin indications at a pediatric medical center to address the shortage. A multidisciplinary task force was formed, comprising faculty members from various fields with expertise in intravenous immune globulin use, the drug in shortage. The task force established tiers based on diagnosis severity, urgency, and supporting evidence quality. Tiers 1, 2, and 3 were approved for urgent use, standard care, and available alternative therapies, respectively. This action minimized adverse effect, demonstrating its effectiveness in managing drug shortages in healthcare facilities (Roth et al., 2024).

According to Donnelly et al. (2021), educating and training healthcare providers about drug shortages and how to manage them can improve patient care during shortages. Increased transparency and communication can also help alleviate shortages. The FDA and manufacturers should provide timely and transparent communication about potential shortages to allow healthcare providers to plan and implement mitigation strategies. Collaboration with pharmacy administration and engagement in the hospital’s pharmacy and therapeutics committee are also recommended. This can help in planning how to use limited drug supplies and determine safe alternatives (Donnelly et al., 2021).

The research by Hemmann et al. (2022) emphasizes the importance of continuous communication between clinical pharmacists and critical care physician leadership, as well as providing detailed medical references and regular face-to-face education to prescribers. It suggests that early awareness of drug shortages and effective communication strategies can reduce the impact of drug shortages on medication errors and clinical outcomes (Hemmann et al., 2022).

According to Kanja et al. (2021), improving vaccine availability in public health facilities requires collaboration across sectors, involving all stakeholders to support immunization. The paper emphasizes the critical need to establish synergistic relationships between national and county governments to effectively streamline responsibilities (Kanja et al., 2021).

### 3.4 Therapeutic importance

To highlight the importance of certain compounds, the primary indications for each drug were compiled in Supplementary Table S3. The drugs in shortage were also divided by ATC Category to understand the impact of the shortage in pediatric care. The ATC classification was attributed according to the ATC/DDD Index 2024 (Chen, 2017) aligned with the main indication provided.

To determine the primary therapeutic classifications outlined in Supplementary Table S4, we have used the WHO Model List of Essential Medicines—23rd, 2023 (World Health Organization, 2023a) and the WHO Model List of Essential Medicines for Children—9th, 2023 (World Health Organization, 2023b) as key references. We have also consulted supplementary literature sources (StatPearls, 2024; Drug Bank, 2024).

After comparing both lists, we have found that most of the ingredients are the same in both lists. However, there are some exceptions, including amiodarone, melphalan, fludarabine, cladribine, docetaxel, and fentanyl. These ingredients are only included in the WHO Model List of Essential Medicines - 23rd, 2023, and not in the WHO Model List of Essential Medicines for Children—9th, 2023. Out of 112 substances, approximately 68% (76/112) are present in the WHO Model List of Essential Medicines - 23rd, 2023, and 62% (70/112) are present in the WHO Model List of Essential Medicines for Children—9th, 2023.

The data shows that the antineoplastic agents has had a significant impact in terms of shortages. Antibiotics and vaccines have also been affected, closely following behind. It is important to note that the shortages have impacted a broad range of pediatric treatments across a wide range of diseases.

The medicines most frequently affected by shortages vary significantly across geographic areas, features of National Healthcare Systems, and GDP. The relationship between Gross Domestic Product (GDP) and healthcare systems is crucial in understanding healthcare resource allocation and access to medicines. Countries with higher GDP tend to have more robust healthcare systems, which are better equipped to manage resources and reduce shortages. The features of National Healthcare Systems also play a role, with more developed systems generally having more resources available (Improving access to medicines in, 2012; Healthcare expenditure statistics, 2024).

The data in Supplementary Table S1 shows that higher-income countries in Europe and North America have similar shortages of certain drugs, with antineoplastic agents and antibiotics being the most affected in both cases. The availability of data on medicine shortages is limited in low- and middle-income countries compared to high-income nations. Even so, low-income countries in Africa, Asia, and South America showed that the prevalent drugs in shortage were vaccines such as polio, measles-rubella, Bacille Calmette-Guérin, and others.

## 4 Limitations

There are several limitations to this study that should be noted. Firstly, the manuscript focused only on reviewing literature data, and did not include potentially relevant information provided by Regulatory Agency portals (e.g., guidelines) or National shortages lists. This may have led to important data on medicine shortages and mitigation strategies being omitted.

Another key limitation of this study is that the mitigation strategies identified in the literature were not all empirically tested to determine their effectiveness, therefore there is a lack of quantitative results. Instead of actually testing the suggested methods, they primarily focused on describing the strategies without providing empirical data to support their effectiveness. Without data demonstrating the real-world impact of the mitigation strategies, it is difficult to draw firm conclusions about their utility and recommend them with confidence.

Moreover, the reviewed articles also lacked practical details on how to implement the strategies. They did not provide enough information about the additional effort, resources, and time needed for successful implementation. Having this information would enable a more informed evaluation of the trade-offs and potential barriers and to assess feasibility and cost-effectiveness associated with adopting the suggested strategies, ultimately facilitating more effective decision-making.

Additionally, different countries have varying definitions of medicine shortages based on their regulatory frameworks. Consequently, the effectiveness and applicability of reported mitigation strategies are influenced by the specific regulatory framework in each country. What works well in one country may not be as effective in another due to regulatory differences.

Finally, the study did not conduct a formal quality assessment of the included studies. As a result, it is unclear how reliable the evidence supporting the reported mitigation strategies is. Some strategies may be based on low-quality evidence, which could limit their reliability and applicability.

Despite its limitations, this study offers a thorough review of the literature on medicine shortages and mitigation strategies in pediatric care. The findings can be used as a starting point for further research and to guide the development of policies and interventions to address this significant public health issue.

To address these shortcomings, future research should prioritize empirically testing the mitigation strategies identified in this review, as well as evaluating quantitative and qualitative data on the implementation requirements. Comparative studies that evaluate the impact of different approaches in similar regulatory contexts would help clarify which strategies are most effective. Additionally, including data from regulatory sources and national shortage lists could provide a more comprehensive understanding of the issue and potential solutions.

By addressing these limitations, subsequent studies can build upon the findings of this review and offer more definitive guidance for policymakers and practitioners seeking to mitigate medicine shortages. Only by filling these gaps in the literature can we develop a more comprehensive understanding of effective strategies for mitigating strategies of drug shortages in pediatric care.

## 5 Conclusion

To effectively address the challenges posed by drug shortages, it is imperative to adopt a comprehensive risk management approach that takes into account various factors. This approach must be multi-faceted, with emphasis on prevention through collaboration among international governance, regulatory bodies, both national and local authorities, as well as health workers to forecast and monitor medicine supply. This can be achieved by investing in technology and data analytics that can identify potential shortages early on, enabling healthcare providers to take proactive measures to mitigate their impact.

In addition, it is important to establish institutional and national guidelines that provide a framework for dealing with drug shortages. These guidelines should be developed in collaboration with all stakeholders, including healthcare providers, regulators, and patients. Communication is also a key element, as it can help identify potential shortages and facilitate the sharing of information about alternative treatment options.

Furthermore, education for healthcare providers is crucial in ensuring that they are equipped with the knowledge and skills needed to manage drug shortages effectively. This can include training on how to identify and report shortages, as well as education on alternative treatment options and efficient drug utilization practices.

Another important element of a comprehensive risk management approach is minimizing waste and promoting efficient drug utilization. This can be achieved by implementing strategies such as dose optimization, inventory management, and expiration date tracking. On the other hand, compounding formulations proved to be a crucial strategy. It allows customization of medicines by converting adult formulations into child-friendly forms or altering available products to create personalized medications not readily available, commonly used in hospitals to ensure patients receive necessary medications despite shortages.

In summary, a proactive and collaborative strategy that tackles the underlying causes of drug shortages, improves prevention and monitoring, and is based on communication and education is necessary to ensure the safety and wellbeing of pediatric patients. Such an approach requires a concerted effort and a willingness to invest in the necessary resources and infrastructure to make it a reality. Overall, understanding the geographical and systemic factors contributing to medicine shortages is also crucial for developing effective strategies to mitigate their impact on healthcare services.

## Supplementary material

SUPPLEMENTARY FIGURE S1 PRISMA Flowchart of the search method (15).

SUPPLEMENTARY TABLE S1 Paediatric drug shortages, time interval, and location of each study.

SUPPLEMENTARY TABLE S2 Paediatric drug shortages and proposed handling strategies. Used abbreviations: IV for intravenous, PO for per os, P for parenteral.

SUPPLEMENTARY TABLE S3 Therapeutic classification and importance of the investigated ingredients.

SUPPLEMENTARY TABLE S4 Therapeutic classification and importance of the investigated ingredients.
