# Supplementary material for: Managing drug shortages in pediatric care
Source: Front Pharmacol. 2024 Jun 25;15:1416029. doi: 10.3389/fphar.2024.1416029 (PMC11231388; doi:10.3389/fphar.2024.1416029)
Supplement: Supplementary file 1 [file DataSheet1.docx]

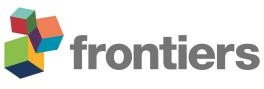


***Supplementary Material***

Managing Drug Shortages in Pediatric Care

# Alexandra Rosário^1^, Balázs Hankó^2^, Romána Zelkó^2*^

*** Correspondence:** Romána Zelkó: [zelko.romana@semmelweis.hu](mailto:zelko.romana@semmelweis.hu)

**Supplementary Table S1** – Paediatric drug shortages, time interval, and location of each study.

| **Shortage Drug(s)** | **Time**  **Frame** | **Location and Settings** | **Reference** |
| --- | --- | --- | --- |
| erwinia asparaginase | 2017 | Single-center retrospective analysis at Cohen Children's Medical Center in New  York, USA. | (16) |
| derivates of penicillin | 2017 - 2018 | Retrospective study on public maternity hospitals in Fortaleza, Brazil, analyzing medical charts from maternal and infant cases | (17) |
| morphine | 2017 - 2018 | Retrospective chart review of postoperative patients in the cardiac intensive care unit at Children's National Hospital in Washington, USA. | (18) |
| daunorubicin, doxorubicin, cytarabine, methotrexate, vincristine, asparaginase, thiotepa | 2014 | Survey by Children’s Oncology Group on chemotherapy shortages' impact on clinical trials and patient care in USA pediatric institutions. | (19) |
| Bacille Calmette-Guerin vaccine | 2015 | Ptbnet web-based survey in pulmonary and tuberculosis specialized hospitals, general outpatient clinics, and primary care clinics in European countries. | (20) |
| amoxicillin | 2022 – 2023 | Quasi-experimental analysis conducted at an urban freestanding children’s hospital system and affiliated community health center in Boston, USA. | (21) |
| anti-rabies immunoglobulin, heparin sodium, rocuronium, vecuronium, ondansetron, lorazepam, amikacin, vancomycin, atropine, ketorolac,  furosemide, ciprofloxacin, tobramycin, amiodarone, promethazine | 2001 – 2015 | Analysis of pediatric emergency and critical care settings in the USA using data from the University of Utah Drug Information Services. | (7) |
| diphtheria, tetanus, pertussis, polio, haemophilus B, hepatitis B vaccine, epinephrine, simethicone, measles, mumps, rubella-varicella vaccine,  diphenhydramine, aciclovir, cefaclor, clarithromycin, pneumococcal conjugate vaccine, lidocaine + epinephrine, cefpodoxime, acetaminophen, dexamethasone, methylphenidate, haemophilus B vaccine, oxycodone, hepatitis A vaccine,  ciprofloxacin | 2001 – 2015 | Data analysis from the University of Utah Drug Information Services on general ambulatory pediatrics settings in the USA. | (6) |
| morphine, lorazepam, dopamine, dexamethasone, levothyroxine, metoclopramide, meropenem, phenobarbital, fosphenytoin, albuterol,  budesonide, aminophylline, erythromycin, naloxone, fluconazole,  alprostadil, diazepam, indomethacin, midazolam, phenylephrine, caffeine citrate, vancomycin, cefotaxime, gentamicin, ampicillin, hydrocortisone | 2001 – 2016 | Data analysis from the University of Utah Drug Information Services on neonatal intensive care units in the USA. | (22) |
| sodium bicarbonate | 2017 | Retrospective chart review at Alberta Children's Hospital and Stollery Children's  Hospital in Alberta, Canada using electronic records. | (23) |
| sodium bicarbonate | 2017 | Retrospective cohort study at Children’s Hospital Colorado, USA, using  electronic medical record chart review. | (24) |
| daunorubicin | 2009 – 2013 | Retrospective cohort study at Aflac Cancer and Blood Disorders Center,  Children's Healthcare of Atlanta, USA, using medical record chart review. | (25) |
| intravenous immunoglobulin | 2019 | Data sourced from the electronic health record system on a tertiary care pediatric  medical center in Washington, USA | (26) |
| trophamine | 2017 – 2018 | Retrospective cohort study at two neonatal intensive care units in Saint Louis  University Hospital, Missouri, USA. | (27) |

| fentanyl, midazolam, lorazepam, diazepam | 2011 – 2012 | Retrospective chart review of patient records at C.S. Mott Children’s Hospital,  University of Michigan, and Texas Children’s Hospital in the USA. | (29) |
| --- | --- | --- | --- |
| polio, measles-rubella, Bacille Calmette-Guerin, rotavirus, diphtheria, haemophilus B, hepatitis B, and pneumococcal vaccines | 2020 | Descriptive cross-sectional study using a questionnaire to collect data at public  health facilities in Nairobi City County, Kenya. | (30) |
| influenza vaccines | 2017 | Demographic data was collected from the Department of Statistics in Taiwan. | (31) |
| sulfamethoxazole + trimethoprim, ranitidine, piperacillin + tazobactam, ciprofloxacin, fluconazole, aciclovir, ondansetron, benzylpenicillin, colistin, phenoxymethylpenicillin, meropenem, amikacin,  cytomegalovirus immunoglobulin, ursodeoxycholic acid, alizapride, clonazepam, dexamethasone, nystatin, pantoprazole, methotrexate,  melphalan, carboplatin, cyclophosphamide, etoposide, busulfan | 2011 – 2017 | Retrospective analysis at Ghent University Hospital, Belgium, using pharmacy data and manual follow-up notes. | (32) |
| sodium bicarbonate | 2017 | Retrospective chart review at Texas Children’s Hospital Cancer Center, USA,  using patients' electronic medical records. | (8) |
| carboplatin, cisplatin, cytarabine, doxorubicin, vincristine | 2018 – 2020 | Thematic analysis from five Caribbean hospitals using qualitative interviews,  literature, and policy documents. | (33) |
| haemophilus B vaccine | 2023 | Data analyzed from Merative™ MarketScan® Research Databases and the  National Immunization Survey-Child in the USA. | (34) |
| cefotaxime | 2015 – 2017 | Single-center retrospective cohort study at NICU, University of Chicago  Medicine Comer Children’s Hospital, USA. | (35) |
| methotrexate, carmustine, doxorubicin, cisplatin, amsacrine, etoposide, tioguanine, mercaptopurine, cytarabine, cladribine, cyclophosphamide, vincristine, carboplatin, procarbazine, dactinomycin, vindesine,  bleomycin, hydroxycarbamide, busulfan, asparaginase, idarubicin | 2001 – 2014 | Single-center retrospective study at a Belgian tertiary care hospital. | (36) |
| favipiravir | 2022 | Chulabhorn Hospital in Bangkok, Thailand. | (37) |
| diphtheria vaccine, measles containing vaccine, bacille Calmette-Guérin vaccine, oral polio vaccine | 2023 | Data from WHO/UNICEF on Immunization used for a retrospective cohort study  in 131 countries across various regions (America, Eastern Mediterranean, Europe, South-East Asia, Western Pacific). | (5) |
| bacille Calmette-Guérin vaccine, pneumococcal vaccine, oral polio  vaccine, yellow fever vaccine | 2023 | Phone interviews with health providers collected data on vaccination coverage  and clinical outcomes in 156 health facilities in Western North Region, Ghana. | (29) |
| trofosfamide, cytarabine, etoposide, melphalan, vindesine, lomustine, clofarabine, all-trans retinoic acid, thiotepa, everolimus, pamidronate, docetaxel, temozolomide, fludarabine, idarubicin, dexamethasone, asparaginase, dactinomycin, methotrexate, bleomycin, daunorubicin,  carboplatin | 2016 - 2018 | Survey distributed to health professionals in 34 European countries and parents from 20 European countries. | (30) |

**Supplementary Table S2 –** Paediatric drug shortages and proposed handling strategies. Used abbreviations: IV for intravenous, PO for per os, P for parenteral.

| **Shortage Drug(s)** | **Dosage Form** | **Handling the Shortage** | **Reference** |
| --- | --- | --- | --- |
| erwinia asparaginase | Not indicated | Universal premedication and drug monitoring for asparaginase to prevent adverse events and drug substitutions; pegylated E. coli derived  asparaginase desensitization. | (16) |
| derivates of penicillin | IV | Treatment with other therapeutic schemes; search for alternatives;  ceftriaxone and cefazolin as substitutes. | (17) |
| morphine | IV | Use adjuvant pain medications like acetaminophen and non-steroidal  anti-inflammatory drugs; fentanyl and hydromorphone as substitutes; opioid stewardship program; IV to PO conversion; provider education. | (18) |
| daunorubicin, doxorubicin, cytarabine, methotrexate, vincristine,  asparaginase, thiotepa | Not indicated | Obtain drugs externally; convert forms; cohort patients; modify  treatments; reduce dosages; issue guidelines. | (19) |
| Bacille Calmette-Guerin vaccine | Not indicated | Prioritize high-risk individuals; cohort vaccinees; change suppliers; stockpile vaccines; collaboration between national health agencies and  manufacturers. | (20) |
| amoxicillin | Liquid Suspension | Amoxicillin-clavulanate and cefdinir as substitutes; split tablets or  capsules; use second-line agents; practice watchful waiting. | (21) |
| anti-rabies immunoglobulin, heparin sodium, rocuronium, vecuronium, ondansetron, lorazepam, amikacin, vancomycin, atropine, ketorolac, furosemide, ciprofloxacin, tobramycin,  amiodarone, promethazine | Not indicated | Collaboration with pharmacy administration and engagement in the hospital's pharmacy and therapeutics committee; increased transparency and communication; improved quality control; diversification of  manufacturing sources; incentives for manufacturers; stockpiling of  critical drugs; education and training. | (7) |
| diphtheria, tetanus, pertussis, polio, haemophilus B, hepatitis B vaccine, epinephrine, simethicone, measles, mumps, rubella- varicella vaccine, diphenhydramine, aciclovir, cefaclor,  clarithromycin, pneumococcal conjugate vaccine, lidocaine + epinephrine, cefpodoxime, acetaminophen, dexamethasone,  methylphenidate, haemophilus B vaccine, oxycodone, hepatitis A vaccine, ciprofloxacin | P: DTaP/IPV/Hib/HepB vaccine, epinephrine, MMRV vaccine, diphenhydramine, PCV7 vaccine, lidocaine +  epinephrine, dexamethasone, Hib vaccine, HepA vaccine; PO: Tablet - simethicone, acyclovir, methylphenidate,  clarithromycin, acetaminophen, ciprofloxacin; Suspension – cefaclor, cefpodoxime; Oral solution - oxycodone | Create critical pediatric drug list; ensure equitable distribution; monitor shortages; identify alternatives; early manufacturer  notifications; support research. | (6) |
| morphine, lorazepam, dopamine, dexamethasone, levothyroxine, metoclopramide, meropenem, phenobarbital, fosphenytoin,  albuterol, budesonide, aminophylline, erythromycin, naloxone, fluconazole, alprostadil, diazepam, indomethacin, midazolam, phenylephrine, caffeine citrate, vancomycin, cefotaxime,  gentamicin, ampicillin, hydrocortisone | P: morphine, lorazepam, dopamine, dexamethasone, levothyroxine  e, metoclopramide, meropenem, phenobarbital, fosphenytoin, alprostadil, fluconazole, aminophylline,  naloxone; I: albuterol, budesonide; O: erythromycin; Not indicated: indomethacin, midazolam, phenylephrine,  caffeine citrate, vancomycin, cefotaxime, gentamicin, ampicillin, hydrocortisone | Anticipate shortages; import drugs; prioritize vulnerable patients; use pediatric formulations for these patients only; administer the same  medications at the same time; follow entities guidelines; avoid hoarding; consider alternative formulations; daily treatment assessment; drug  compounding; review available products; flexible supply strategies;  expand suppliers; use expired meds with approval; evaluate replacement medications. | (22) |
| sodium bicarbonate | IV | Use oral NaHCO3, IV Lactated Ringer’s for oral urine alkalinization. | (23) |
| sodium bicarbonate | IV | Use alternative agents for oral urine alkalinization. | (24) |
| daunorubicin | IV | Substitute daunorubicin for mitoxantrone. | (25) |
| intravenous immunoglobulin | IV | Multidisciplinary task force and tier system. | (26) |

| trophamine | Parenteral | Reduce trophamine use and fortify early; adjust feeding guidelines. | (11) |
| --- | --- | --- | --- |
| fentanyl, midazolam, lorazepam, diazepam | IV | Use alternatives; communication between pharmacist and prescribers;  patient prioritization; standardized conservation approach. | (28) |
| polio, measles-rubella, Bacille Calmette-Guerin, rotavirus, diphtheria, haemophilus B, hepatitis B, and pneumococcal vaccines | PO: polio; Not indicated: measles-rubella, Bacille Calmette-Guerin, rotavirus, diphtheria,  haemophilus B, hepatitis B, and pneumococcal vaccines | Enhance vaccine delivery planning; train supply chain officers; improve forecasting and transportation efficiency; collaboration between  stakeholders. | (29) |
| influenza vaccines | Injectable | Sharing and redistributing influenza vaccines. | (30) |
| sulfamethoxazole + trimethoprim, ranitidine, piperacillin + tazobactam, ciprofloxacin, fluconazole, aciclovir, ondansetron,  benzylpenicillin, colistin, phenoxymethylpenicillin, meropenem, amikacin, cytomegalovirus immunoglobulin, ursodeoxycholic acid,  alizapride, clonazepam, dexamethasone, nystatin, pantoprazole, methotrexate, melphalan, carboplatin, cyclophosphamide,  etoposide, busulfan | P: piperacillin/tazobactam, ciprofloxacin, aciclovir,  benzylpenicillin, meropenem, amikacin, cytomegalovirus immunoglobulin, ursodeoxycholic acid, alizapride,  clonazepam, potassium phosphate, methotrexate,  melphalan, carboplatin, cyclophosphamide, etoposide, busulfan; PO: sulfamethoxazol/trimethoprim, ranitidine, fluconazol, ondansetron, phenoxymethylpenicillin,  dexamethasone, nystatin, pantoprazole | Purchase generics; import urgently; pharmaceutical modifications or preparation in-house; cohort patients; change of ongoing treatments. | (31) |
| sodium bicarbonate | IV | Revise institutional High-Dose Methotrexate guidelines; consider  alternative IV fluid compositions for hydration and urine alkalization. | (32) |
| carboplatin, cisplatin, cytarabine, doxorubicin, vincristine | Not indicated | Expand National Lists of Essential Medicines for childhood cancer meds;  improve supply chain; substitute formulations; needs-based forecasting. | (8) |
| haemophilus B vaccine | Injectable | Delaying vaccine booster in favor of primary series doses. | (33) |
| cefotaxime | Injectable | Use ceftazidime as an alternative. | (34) |
| methotrexate, carmustine, doxorubicin, cisplatin, amsacrine, etoposide, tioguanine, mercaptopurine, cytarabine, cladribine, cyclophosphamide, vincristine, carboplatin, procarbazine,  dactinomycin, vindesine, bleomycin, hydroxycarbamide, busulfan, asparaginase, idarubicin | Mostly injectable | Buy generics; adjust individual treatment plans; cohort  patients, import drugs; obtain drugs from another institution; seek alternative suppliers. | (35) |
| favipiravir | PO | Formulation of favipiravir into a child-friendly oral solution. | (36) |
| diphtheria vaccine, measles containing vaccine, bacille Calmette- Guérin vaccine, oral polio vaccine | Not indicated | Improving supply chain systems; Global Vaccine Action Plan 2011–  2020; Immunization Agenda 2030. | (37) |
| bacille Calmette-Guérin vaccine, pneumococcal vaccine, oral polio  vaccine, yellow fever vaccine | Not indicated | Utilize aerial logistics for enhanced supply-chain logistics. | (5) |
| trofosfamide, cytarabine, etoposide, melphalan, vindesine, lomustine, clofarabine, all-trans retinoic acid, thiotepa, everolimus,  pamidronate, docetaxel, temozolomide, fludarabine, idarubicin,  dexamethasone, asparaginase, dactinomycin, methotrexate, bleomycin, daunorubicin, carboplatin | Not indicated | Legislation for early shortage alerts; European Union strategic plans; production incentives; national essential lists; development of a national database on shortages; enhanced procurement models. | (11) |

**Supplementary Table S3 –** Therapeutic classification and importance of the investigated ingredients.

| Strategy Class | Key Points | Outcomes |
| --- | --- | --- |
| Drug Supply and Distribution | - Seeking alternative sources and giving financial incentives to suppliers  - Obtaining missing drugs from other institutions | - Improves drug distribution processes  - Enables access to alternative vial sizes |
| Therapeutic Alternatives | - Using substitutes or adapted therapies, particularly for less vulnerable patients | - Maintains effective therapy when standard treatment is unavailable  - Ensures access for high-risk groups during limited availability |
| Compounding Pharmaceutical Formulations | - Compounding adult forms into child-friendly forms  - Converting missing drug to another form | - Provides personalized medications not readily available  - Offers potential solutions for specific dosage needs |
| Inventory Management and Waste Reduction | - Avoiding stockpiling or excessive purchases  - Carefully reviewing available products  - Treating patients requiring same medication simultaneously or within shelf-life | - Reduces drug waste  - Ensures availability of critical drugs during shortages |
| Patient cohorting and Prioritization | - Prioritizing most critically ill patients  - Cohorting patients for specific parenteral treatments | - Mitigates shortages at the hospital level  - Ensures access for high-risk groups during limited availability |
| Forecasting and Shortage Monitoring | - Identifying factors causing shortages  - Implementing real-time monitoring and alerts | - Enables proactive mitigation before shortages occur  - Allows for greater transparency and quicker identification of potential shortages |
| Guidelines and Policies | - Collaboration between national agencies, manufacturers, providers, pharmacists, and physician leadership  - Educating providers on drug shortages and management strategies  - Transparent communication about potential shortages | - Ensures patients receive best possible care during shortages  - Improves planning and implementation of mitigation strategies  - Reduces impact on medication errors and clinical outcomes |

**Supplementary Table S4 –** Therapeutic classification and importance of the investigated ingredients.

| **ATC Category** | **Substance in**  **Shortage** | **WHO**  **EML** | **WHO**  **EMLc** | **Main Indication** |
| --- | --- | --- | --- | --- |
| **A02 Drugs for acid- related disorders** | ranitidine | Yes | Yes | Gastroesophageal reflux disease; Peptic ulcer disease; Zollinger-Ellison syndrome (28). |
|  | pantoprazole | No | No | Gastroesophageal reflux disease; Zollinger-Ellison  syndrome; Erosive esophagitis (28). |
| **A03 Drugs for functional gastrointestinal disorders** | simethicone | No | No | Flatulence (28). |
|  | atropine | Yes | Yes | Organophosphate/muscarinic poisoning; Bradycardia (28). |
|  | metoclopramide | Yes | Yes | Vomiting and nausea associated with gastroesophageal  reflux disease, chemotherapy or certain surgical or diagnostic procedures (28). |
|  | alizapride | No | No | Vomiting and nausea associated with medical  procedures, surgeries, and cancer therapies (29) |
| **A04 Antiemetics and antinauseants** | ondansetron | Yes | Yes | Nausea and vomiting following surgery, chemotherapy, or radiation therapy (28). |
| **A05 Bile and liver therapy** | ursodeoxycholic acid | No | No | Gallstone disease, primary biliary cholangitis, primary  sclerosing cholangitis, intrahepatic cholestasis of pregnancy, and bile reflux gastrites (28). |
| **A06 Drugs for**  **constipation** | naloxone | Yes | Yes | Accidental or intentional overdose, acute or chronic  toxicity from opioids (33). |
| **B01 Antithrombotic agents** | heparin sodium | Yes | Yes | Prophylaxis and treatment of venous thrombosis; conditions requiring anticoagulant therapy (28). |
| **B05 Blood substitutes**  **and perfusion solutions** | sodium  bicarbonate | No | No | Alkalinize urine with intravenous hydration for  adequate renal clearance during HDMTX (10) |
| **C01 Cardiac therapy** | amiodarone | Yes | No | Ventricular arrhythmias (28). |
|  | dopamine | Yes | Yes | Chronic congestive heart failure; trauma; renal failure; open-heart surgery; shock from myocardial infarction  or septicemia (28). |
|  | phenylephrine | No | No | Hypotension (28). |
|  | epinephrine | Yes | Yes | Type 1 hypersensitivity reactions (28). |
|  | alprostadil | No | No | Erectile dysfunction; ductus arteriosus in newborns with congenital heart diseases (28). |
| **C03 Diuretics** | furosemide | Yes | Yes | Volume overload and edema secondary to congestive  heart failure exacerbation, liver failure, or renal failure (28). |
| **G01 Gynecological**  **antiinfectives and antiseptics** | nystatin | Yes | Yes | Cutaneous, mucocutaneous, and oral *Candida spp.*  Infections (28). |
| **H02 Corticosteroids for systemic use** | dexamethasone | Yes | Yes | Multiple sclerosis; allergies; cerebral edema;  inflammation; shock; asthma; atopic and contact dermatitis; drug hypersensitivity reactions (28). |
|  | hydrocortisone | Yes | Yes | Dermatose; endocrine disorders; immune conditions; allergic disorders (29) |
| **H03 Systemic hormonal preparations, excluding sex hormones and**  **insulins** | levothyroxine | Yes | Yes | Primary, secondary, and tertiary hypothyroidism (28). |
| **J01 Antibacterials for systemic use** | amoxicillin | Yes | Yes | Community acquired pneumonia (mild to moderate and severe); Complicated severe acute malnutrition; Otitis media; Pharyngitis; Progressive apical dental abscess; Sepsis in neonates and children; Sinusitis;  Uncomplicated severe acute malnutrition (30,31). |
|  | ampicillin | Yes | Yes | Community acquired pneumonia (severe); Complicated intraabdominal infections; Complicated  severe acute malnutrition; Sepsis in neonates and children (30,31). |
|  | gentamicin | Yes | Yes | Acute bacterial meningitis in neonates; Community acquired pneumonia (severe); Complicated intraabdominal infections; Complicated severe acute malnutrition; Sepsis in neonates and children (30,31). |

|  | benzylpenicillin | Yes | Yes | Community acquired pneumonia (severe); Complicated severe acute malnutrition; Sepsis in neonates and children; Syphilis (congenital) (30,31). |
| --- | --- | --- | --- | --- |
|  | phenoxymethylpeni  cillin | Yes | Yes | Community acquired pneumonia (mild to moderate);  Pharyngitis; Progressive apical dental abscess (30,31). |
|  | piperacillin + tazobactam | Yes | Yes | Complicated intraabdominal infections (severe); High- risk febrile neutropenia; Hospital acquired pneumonia;  Necrotizing fasciitis (30,31). |
|  | cefaclor | No | No | Bacterial infections, including pneumonia, ear infections, lung infections, skin infections, throat  infections, and urinary tract infections (29). |
|  | cefpodoxime | No | No | Mild to moderate bacterial infections, including gonorrhea, community acquired pneumonia, and  sinusitis (29). |
|  | meropenem | Yes | Yes | Acute bacterial meningitis in neonates; Complicated intraabdominal infections (severe); High-risk febrile  neutropenia (30,31). |
|  | sulfamethoxazole + trimethoprim | Yes | Yes | Lower urinary tract infections; Acute invasive bacterial diarrhea/dysentery (30,31). |
|  | erythromycin | Yes | Yes | Infections,caused by bactéria, such as Chlamydia trachomatis or Neisseria gonorrhoeae (30,31). |
|  | clarithromycin | Yes | Yes | Community acquired pneumonia (severe); pharyngitis (30,31) |
|  | tobramycin | No | No | Various infections, including gram-negative bacteria and Staphylococcus aureus infections, such as septicemia, lower respiratory tract infections,  meningitis, intra-abdominal infections, skin infections, osteomyelitis, and urinary tract infections (28). |
|  | amikacin | Yes | Yes | High-risk febrile neutropenia; Pyelonephritis (severe);  Tuberculosis (30,31). |
|  | ciprofloxacin | Yes | Yes | Acute invasive bacterial diarrhea/dysentery; Enteric fever; Low-risk febrile neutropenia; Pyelonephritis  (mild to moderate) (30,31). |
|  | vancomycin | Yes | Yes | Endophthalmitis; Necrotizing fasciitis (30,31). |
|  | colistin | Yes | Yes | Acute or chronic infections due to sensitive strains of certain gram-negative bacilli, particularly  Pseudomonas aeruginosa (29). |
| **J02 Corticosteroids for systemic use** | fluconazole | Yes | Yes | Vaginal candidiasis; oropharyngeal and esophageal candidiasis; peritonitis; systemic Candida infections  (28). |
| **J05**  **Immunosuppressants** | aciclovir | Yes | Yes | Infections caused by HSV (28). |
|  | favipiravir | No | No | Infections caused by influenza virus, Ebola vírus and COVID-19 (24) |
| **J06 Immune sera and**  **immunoglobulins** | intravenous  immunoglobulin | Yes | Yes | Replacement therapy for antibody deficiencies, such  as primary immunodeficiency diseases (29). |
|  | cytomegalovirus immunoglobulin | No | No | Cytomegalovirus infections (29). |
|  | anti-rabies  immunoglobulin | Yes | Yes | Rabies (30,31). |
| **J07 Vaccines** | haemophilus B vaccine | Yes | Yes | Haemophilus influenzae type B (30,31) |
|  | pneumococcal vaccine | Yes | Yes | Pneumococcal disease caused by specific serotypes of Streptococcus pneumoniae (30,31). |
|  | bacille Calmette-  Guérin vaccine | Yes | Yes | Tuberculosis (5) |
|  | diphteria vaccine | Yes | Yes | Diphtheria (30,31). |
|  | measles vaccine | Yes | Yes | Measles (30,31) |
|  | influenza vaccine | Yes | Yes | Infections by influenza virus (17,30,31) |
|  | Hepatitis A vaccine | Yes | Yes | Hepatitis A (30,31) |
|  | measles, mumps,  rubella-varicella vaccine | No | No | Measles; Mumps; Rubella; Varicella (30,31) |

|  | measles-rubella vaccine | No | No | Measles; Rubella (30,31). |
| --- | --- | --- | --- | --- |
|  | poliomyelitis vaccine | Yes | Yes | Poliomyelitis (30,31). |
|  | rotavirus vaccine | Yes | Yes | Infections by rotavirus (30,31). |
|  | yellow fever  vaccine | Yes | Yes | Yellow fever (30,31). |
| **L01 Antineoplastic agents** | cyclophosphamide | Yes | No | Acute lymphoblastic leukemia; Anaplastic large cell lymphoma; Burkitt lymphoma; Diffuse large B-cell lymphoma; Ewing sarcoma; Hodgkin lymphoma; Low-grade glioma; Nephroblastoma (Wilms tumor);  Rhabdomyosarcoma (30). |
|  | melphalan | Yes | No | Multiple myeloma (30). |
|  | lomustine | No | No | Primary and metastatic brain tumors (29) |
|  | trofosfamide | No | No | Ependymomas; Medulloblastomas; Sarcoma; Soft  Tissue; Supratentorial PNETs; Recurrent Brain Tumors (29) |
|  | busulfan | No | No | Chronic myeloid leucemia (28) |
|  | thiotepa | No | No | Breast adenocarcinoma; Ovary adenocarcinoma; Superficial papillary bladder cancer (29) |
|  | carmustine | No | No | Astrocytoma; Brainstem glioma; Ependymoma;  Glioblastoma; Medulloblastoma (29) |
|  | methotrexate | Yes | Yes | Acute lymphoblastic leukaemia; Acute promyelocytic leukemia; Anaplastic large cell lymphoma; Burkitt lymphoma; Langerhans cell histocytosis;  Osteosarcoma (31). |
|  | mercaptopurine | Yes | Yes | Acute lymphoblastic leukaemia; Acute promyelocytic  leukaemia; Langerhans cell histocytosis (30,31). |
|  | all-trans retinoic acid | Yes | Yes | Acute promyelocytic leukaemia (30,31). |
|  | hydroxycarbamide | Yes | Yes | Haemoglobinopathies; Chronic myeloid leukaemia  (30,31). |
|  | fludarabine | Yes | No | Chronic lymphocytic leukaemia (30,31). |
|  | tioguanine | Yes | Yes | Acute lymphoblastic leukaemia (30,31). |
|  | temozolomide | No | No | Glioblastoma multiforme; Refractory anaplastic astrocytoma (29). |
|  | cladribine | Yes | No | Leukemia; Multiple sclerosis (28) |
|  | clofarabine | No | No | Relapsed or refractory acute lymphoblastic leucemia  (29) |
|  | cytarabine | Yes | Yes | Acute lymphoblastic leukaemia; Acute myeloid leukaemia; Acute promyelocytic leukaemia; Anaplastic large cell lymphoma; Burkitt lymphoma;  Langerhans cell histiocytosis (30,31). |
|  | vincristine | Yes | Yes | Acute lymphoblastic leukaemia; Burkitt lymphoma; Diffuse large B-cell lymphoma; Ewing sarcoma; Hodgkin lymphoma; Kaposi sarcoma; Langerhans cell histiocytosis; Low-grade glioma; Nephroblastoma (Wilms tumour); Retinoblastoma; Rhabdomyosarcoma  (31). |
|  | vindesine | No | No | Acute lymphocytic leukemia (29) |
|  | etoposide | Yes | Yes | Acute lymphoblastic leukaemia; Acute myeloid leukaemia; Anaplastic large cell lymphoma; Burkitt lymphoma; Ewing sarcoma; Hodgkin lymphoma; Nephroblastoma (Wilms tumour); Osteosarcoma; Ovarian germ cell tumours; Retinoblastoma;  Testicular germ cell tumours (31). |
|  | docetaxel | Yes | No | Early-stage breast cancer; Metastatic breast cancer;  Metastatic prostate cancer (30). |
|  | daunorubicin | Yes | Yes | Acute lymphoblastic leukemia; Acute myeloid leukemia; Acute promyelocytic leukemia (30,31). |
|  | doxorubicin | Yes | Yes | Acute lymphoblastic leukemia; Anaplastic large cell lymphoma; Burkitt lymphoma; Diffuse large B-cell lymphoma; Early stage breast cancer; Ewing sarcoma;  Follicular lymphoma; Hodgkin lymphoma; Kaposi sarcoma; Metastatic breast cancer; Multiple myeloma; |

|  |  |  |  | Nephroblastoma (Wilms tumour); Osteosarcoma (30,31). |
| --- | --- | --- | --- | --- |
|  | idarubicin | No | No | Acute myeloid leukemia (29) |
|  | bleomycin | Yes | Yes | Hodgkin lymphoma; Kaposi sarcoma; Ovarian germ cell tumour; Testicular germ cell tumour (30,31). |
|  | cisplatin | Yes | Yes | Cervical cancer; Head and neck cancer (as a radio- sensitizer); Low-grade glioma; Nasopharyngeal cancer (as a radio-sensitizer); Non-small cell lung cancer;  Osteosarcoma; Ovarian germ cell tumour; Testicular germ cell tumour (30,31). |
|  | carboplatin | Yes | Yes | Cervical cancer; Early stage breast cancer; Epithelial ovarian cancer; Head and neck cancer (as a radio- sensitizer); Low-grade glioma; Nasopharyngeal cancer; Nephroblastoma (Wilms tumor); Non-small cell lung cancer; Osteosarcoma; Ovarian germ cell  tumor; Retinoblastoma; Testicular germ cell tumor (30,31). |
|  | amsacrine | No | No | Acute adult leukemia (29) |
|  | dactinomycin | Yes | Yes | Ewing sarcoma; Gestational trophoblastic neoplasia;  Nephroblastoma (Wilms tumor); Rhabdomyosarcoma (30,31). |
|  | everolimus | Yes | Yes | Subependymal giant cell astrocytoma (30,31). |
|  | procarbazine | Yes | Yes | Hodgkin lymphoma (30,31). |
|  | asparaginase | Yes | Yes | Acute lymphoblastic leukaemia (30,31). |
|  | ketorolac | No | No | Moderate to severe pain, rheumatoid arthritis, osteoarthritis, ankylosing spondylitis, menstrual  disorders, and headaches (28) |
| **M01 Anti-inflammatory and antirheumatic products, non-steroids** |  |  |  |  |
|  | indomethacin | No | No | Inflammatory disorders, such as rheumatoid arthritis, gout, bursitis, and tendonitis (28) |
| **M03 Muscle relaxants** | vecuronium | Yes | Yes | Muscle relaxant (30,31) |
|  | rocuronium | No | No | Muscle relaxant (28) |
| **M05 Drugs affecting bone structure and mineralization** | pamidronate | No | No | Moderate or severe hypercalcemia of malignancy; Moderate to severe Paget disease of bone; Osteolytic bone metastases of breast cancer; Osteolytic lesions of  multiple myeloma (28) |
| **N01 Anesthetic drugs** | lidocaine + epinephrine | Yes | Yes | Local pain (30,31) |
| **N02 Analgesics** | acetaminophen | Yes | Yes | Pain and palliative care (30,31) |
|  | fentanyl | Yes | No | Cancer pain (30) |
|  | morphine | Yes | Yes | Sedation for short-term procedures; Analgesic and pain management (30,31) |
|  | oxycodone | No | No | Management of acute or chronic moderate to severe pain (28) |
| **N03 Antiepileptics** | clonazepam | No | No | Acute treatment of panic disorder, epilepsy, and nonconvulsive status epilepticus (28) |
|  | fosphenytoin | No | No | Seizures (28) |
|  | phenobarbital | Yes | Yes | Seizures (30,31) |
|  | diazepam | Yes | Yes | Anxiety disorders and symptoms; Spasticity associated with upper motor neuron disorders; Muscle spasms; Severe recurrent convulsive seizures; Status  epilepticus (28) |
|  | lorazepam | Yes | Yes | Seizures (30,31) |
|  | midazolam | Yes | Yes | Seizures (30,31) |
| **N06 Psychoanaleptics** | caffeine citrate | Yes | Yes | Apnea of prematurity (29). |
|  | methylphenidate | No | No | Deficit hyperactivity disorder (28) |
| **R03 Drugs for obstructive airway diseases** | albuterol | No | No | Acute or severe bronchospasm (28) |
|  | budesonide | Yes | Yes | Asthma (30,31) |
|  | aminophylline | No | No | Reversible airway obstruction due to asthma or other chronic lung diseases (28) |
| **R06 Antihistamines for**  **systemic use** | diphenhydramin | No | No | Dystonias; insomnia; pruritis; urticaria; vertigo;  motion sickness (28) |

|  | promethazine | No | No | Allergic conditions, including seasonal allergic rhinitis and allergic conjunctivitis, uncomplicated skin manifestations of urticaria, and angioedema (28). |
| --- | --- | --- | --- | --- |
| **Not applied** | trophamine | No | No | Nutritional support for patients who are unable to  consume adequate nutrients orally (14) |


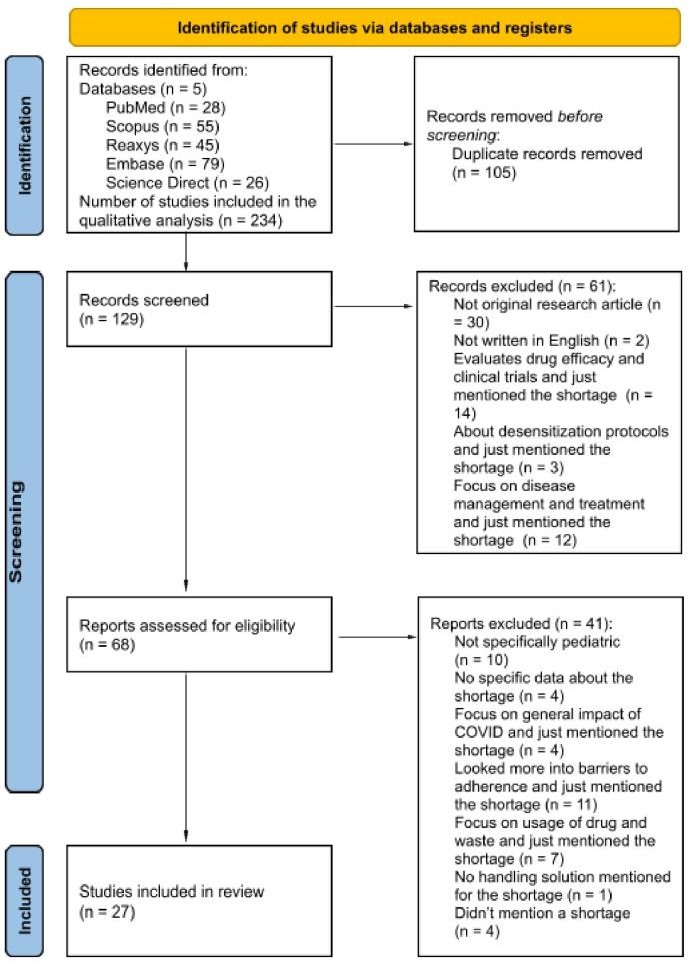


**Supplementary Figure S1.** PRISMA Flowchart of the search method (15).

Supplementary Material

2
